# Effects of pressure-controlled and volume-controlled ventilation on respiratory mechanics and systemic stress response during laparoscopic cholecystectomy

**DOI:** 10.1186/s40064-016-1963-5

**Published:** 2016-03-08

**Authors:** Oznur Sen, Tarik Umutoglu, Nurdan Aydın, Mehmet Toptas, Ayse Cigdem Tutuncu, Mefkur Bakan

**Affiliations:** Department of Anesthesiology and Reanimation, Ministry of Health Haseki Training and Research Hospital, Istanbul, Turkey; Department of Anesthesiology and Reanimation, Bezmialem Vakif University Faculty of Medicine, Vatan Cad, 34093 Fatih, Istanbul, Turkey; Department of Anesthesiology and Reanimation, Istanbul University Cerrahpasa Medical Faculty, Istanbul, Turkey

**Keywords:** Mechanical ventilation, Airway pressure, Systemic stress response, Cortisol, Insulin

## Abstract

Pressure-controlled ventilation (PCV) is less frequently employed in general anesthesia. With its high and decelerating inspiratory flow, PCV has faster tidal volume delivery and different gas distribution. The same tidal volume setting, delivered by PCV versus volume-controlled ventilation (VCV), will result in a lower peak airway pressure and reduced risk of barotrauma. We hypothesized that PCV instead of VCV during laparoscopic surgery could achieve lower airway pressures and reduce the systemic stress response. Forty ASA I-II patients were randomly selected to receive either the PCV (Group PC, n = 20) or VCV (Group VC, n = 20) during laparoscopic cholecystectomy. Blood sampling was made for baseline arterial blood gases (ABG), cortisol, insulin, and glucose levels. General anesthesia with sevoflurane and fentanyl was employed to all patients. After anesthesia induction and endotracheal intubation, patients in Group PC were given pressure support to form 8 mL/kg tidal volume and patients in Group VC was maintained at 8 mL/kg tidal volume calculated using predicted body weight. All patients were maintained with 5 cmH_2_O positive-end expiratory pressure (PEEP). Respiratory parameters were recorded before and 30 min after pneumoperitonium. Assessment of ABG and sampling for cortisol, insulin and glucose levels were repeated 30 min after pneumoperitonium and 60 min after extubation. The P-peak levels observed before (18.9 ± 3.8 versus 15 ± 2.2 cmH_2_O) and during (23.3 ± 3.8 versus 20.1 ± 2.9 cmH_2_O) pneumoperitoneum in Group VC were significantly higher. Postoperative partial arterial oxygen pressure (PaO_2_) values are higher (98 ± 12 versus 86 ± 11 mmHg) in Group PC. Arterial carbon dioxide pressure (PaCO_2_) values (41.8 ± 5.4 versus 36.7 ± 3.5 mmHg) during pneumoperitonium and post-operative mean cortisol and insulin levels were higher in Group VC. When compared to VCV mode, PCV mode may improve compliance during pneumoperitoneum, improve oxygenation and reduce stress response postoperatively and may be more appropriate in patients having laparoscopic surgery.

## Background

Laparoscopic cholecystectomy has virtually replaced classical open cholecystectomy, but the increase in intra-abdominal pressure due to pneumoperitoneum has some consequences. The cardio-pulmonary and metabolic changes during pneumoperitoneum are complex and associated with the level of intra-abdominal pressure (which gives rise to increases in airway pressures), duration of pneumoperitoneum, patient position and the ventilation mode (Marca et al. 1990; Cunningam and Brul 1993).

The most frequently used ventilation mode in general anesthesia is volume-controlled ventilation (VCV), which utilizes a constant flow to deliver a target tidal volume and ensures minute ventilation, may result in high airway pressures in laparoscopic surgery. Pressure-controlled ventilation (PCV), which has been initially proposed in ICU patients with acute respiratory distress syndrome as an alternative to VCV (Davis et al. 1996), is less frequently employed in general anesthesia. With its high and decelerating inspiratory flow, PCV has faster tidal volume delivery and different gas distribution. The same tidal volume setting, delivered by PCV versus VC, will result in a lower peak airway pressure and reduced risk of barotrauma (Davis et al. 1996; Mercat et al. 1993; Campbell and Davis 2002).

We hypothesized that PCV instead of VCV during laparoscopic surgery could achieve lower airway pressures and reduce the systemic stress response. In this study, we have aimed at comparing the effects of PCV and VCV modes during laparoscopic surgery on respiratory mechanics, oxygenation, and hemodynamics, as well as blood cortisol and insulin levels, which has not been investigated before.

## Methods

This prospective randomized controlled study was approved by the Institutional Review Board of Haseki Training and Research Hospital (Number: 131, Date: 18.06.2014, Istanbul, Turkey). The study was carried out according to the principles of declaration of Helsinki and all patients were asked for their signed and informed consent. Forty patients of 18–70 years of age in the ASA I-II risk classification were included in the study. Patients with morbid obesity (BMI exceeding 30 kg/m^2^), history of cardiac, pulmonary, hepato-renal, endocrine, cerebrovascular and neuromuscular diseases or thoracic surgery were excluded. Further, only those cases admitted to the operating theatre until 11:00 A.M. were included in this study. Patients were randomly selected, by opening sealed envelopes, to receive either the PCV (Group PC, n = 20) or VCV (Group VC, n = 20) mode of ventilation during anesthesia.

On arrival at the operating room, standard monitoring was applied consisting of ECG, pulse oximetry and temperature. Intravenous midazolam 0.03 mg/kg was administered. After Allen test and local anesthetic infiltration, a cannula was placed to the radial artery and arterial pressures were monitorized. Blood sampling was made for baseline arterial blood gases (ABG) analysis and for cortisol, insulin and glucose levels to assess the systemic stress response. Anesthesia was induced with propofol 2 mg/kg and fentanyl 2 μg/kg. Rocuronium 0.6 mg/kg was given to facilitate tracheal intubation. Anesthesia was maintained with 0.5–1.0 MAC of sevoflurane in a mixture of oxygen and air (FiO_2_: 50 %). Fentanyl 0.5–1 μg/kg was added to maintain systolic arterial pressure within ±20 % of the baseline value. The ventilator parameters were set as respiratory rate: 12 breaths/min (constant during anesthesia); inspirium time/expirium time: 1/2 and fresh gas flow 1 L/min. Patients in Group PC were given pressure support to form 8 mL/kg tidal volume (pressure support level was adjusted to maintain the same tidal volume during pneumoperitoneum); while Group VC was maintained at 8 mL/kg tidal volume, both were calculated using predicted body weight. All patients were maintained with 5 cmH_2_O positive-end expiratory pressure (PEEP).

After intubation, before pneumoperitoneum, heart rate, arterial pressures, EtCO_2_, peak, plateau and mean airway pressures (P-peak, P-plateau, P-mean respectively) and dynamic compliance (C-dyn) levels were recorded. Pneumoperitonium was created by CO_2_ insufflation and intra-abdominal pressure was maintained at 14 mmHg by means of an automatic insufflator. Thirty minutes after the pneumoperitonium, the respiratory and hemodynamic parameters were recorded again, and sampling for ABG, cortisol, insulin and glucose levels were repeated. Anesthesia was maintained until the end of surgery.

All patients wore anti-embolic stockings and received enoxaparin 40 mg subcutaneous before surgery; tenoxicam 20 mg IV after anesthesia induction; paracetamol 1 g IV after gallbladder extraction; tramadol 100 mg and ondansetron 8 mg before extubation. Skin incisions were infiltrated with 15–20 ml of bupivacaine 0.5 % before closure. Neuromuscular blockade was antagonized with sugammadex 2 mg/kg and tracheal extubation was carried out when the patient was fully awake. Hemodynamic parameters were recorded and blood sampling for ABG, cortisol, insulin and glucose levels were repeated for the last time 60 min after extubation (without supplemental oxygen).

The primary outcome variable was P-peak levels during pneumoperitoneum. The sample size requirement was based on data from a previous retrospective study (Sen et al. 2014) in our institution with 80 patients in which P-peak levels were 24 ± 2.9 cmH_2_O in Group VC and 20 ± 2.7 cmH_2_O in Group PC. Thus, at an alpha risk of 0.05, 20 patients per group would provide 89 % power and detect a 20 % reduction in P-peak levels.

### Statistical analysis

Windows program SPSS 15.0 was used for the statistical analysis of the results. Descriptive statistics were given in terms of numbers and percentages for categorical variables, and in terms of the mean, standard deviation and the median for the numerical variables. Comparison of two independent groups of variables was carried out using the Student T test when meeting the normal distribution criteria, or by the Mann–Whitney U test when these criteria were not met. Relationship between numerical variables was assessed by means of the Spearman Correlation Analysis. The differences between categorical variables were evaluated by the Chi square analysis. Statistical α (alpha) significance level was accepted with the ‘p’ value below 0.05.

## Results

Demographic parameters, pneumoperitoneum time and perioperative hemodynamic values of patients were closely comparable between groups (Table 1). Respiratory parameters during mechanical ventilation are shown in Table 2. Perioperative arterial blood gas analysis, lactate, cortisol, insulin and glucose levels are shown in Table 3. P-peak levels observed before and during pneumoperitoneum in Group VC were significantly higher when compared to Group PC. Before pneumoperitoneum, P-plateau level was higher in Group VC (17.2 ± 3.5 versus 14.8 ± 2.1 cmH_2_O).

**Table 1:** Demographic parameters and perioperative hemodynamic values

	Group PC (n = 20)	Group VC (n = 20)
Age (year)	49.7 ± 10.8	49.0 ± 13.5
Weight (kg)	74.2 ± 9.3	75.1 ± 11.4
Gender (M/F)	7/13	8/12
Pneumoperitoneum time (min)	45.2 ± 21.6	43.4 ± 19.3
HR (beats/min)		
PRE	80.0 ± 15.9	75.6 ± 5.9
PER	90.2 ± 15.7	81.9 ± 12.2
POST	80.8 ± 5.9	84
SAP (mmHg)		
PRE	135.4 ± 16.8	135.7 ± 18.9
PER	125.1 ± 21.0	127.9 ± 23.9
POST	114.4 ± 13.0	117.3 ± 17.0
MAP (mmHg)		
PRE	112.2 ± 17.1	109.9 ± 17.6
PER	102.7 ± 17.1	100.0 ± 20.0
POST	93.6 ± 12.8	93.6 ± 13.1

*HR* heart rate, *SAP* systolic arterial pressure, *MAP* mean arterial pressure, *PRE* preoperative values, *PER* peroperative values (during pneumoperitonium), *POST* postoperative values

**Table 2:** Respiratory parameters during mechanical ventilation

	Group PC (n = 20)	Group VC (n = 20)	*P*
EtCO_2_ (mmHg)			
I	30.2 ± 1.7	31.5 ± 4.9	NS
II	33.1 ± 1.8	33.0 ± 4.2	NS
P-peak (cmH_2_O)			
I	15.0 ± 2.2	18.9 ± 3.8	<0.001
II	20.1 ± 2.9	23.3 ± 3.8	0.007
P-plateau (cmH_2_O)			
I	14.8 ± 2.1	17.2 ± 3.5	0.024
II	19.9 ± 2.6	21.4 ± 3.7	NS
P-mean (cmH_2_O)			
I	8.8 ± 1.0	9.2 ± 1.0	NS
II	10.2 ± 0.8	10.2 ± 1.1	NS
C-dyn (mL/cmH_2_O)			
I	56.7 ± 12.0	42.7 ± 9.8	<0.001
II	38.0 ± 7.0	34.7 ± 6.7	NS

*EtCO*_*2*_ end-tidal carbon dioxide, *P-peak* peak airway pressure, *P-plateau* plateau airway pressure, *P-mean* mean airway pressure, *C-dyn* dynamic compliance, *I* values after entubation, before pneumoperitonium, *II* values during pneumoperitonium, *NS* not significant

*p* < 0.05: statistically significant

**Table 3:** Perioperative arterial blood gas analysis and systemic stress response

	Group PC (n = 20)	Group VC (n = 20)	*P*
PO_2_ (mmHg)			
PRE	89.1 ± 15.6	85.0 ± 15.0	NS
PER	155 ± 32.3	136.3 ± 36.2	NS
POST	98.4 ± 11.8	86.3 ± 11.3	0.002
PCO_2_ (mmHg)			
PRE	37.3 ± 5.6	38.9 ± 5.4	NS
PER	36.7 ± 3.5	41.8 ± 5.4	0.001
POST	37.4 ± 5.6	39.0 ± 5.3	NS
pH			
PRE	7.41 ± 0.04	7.40 ± 0.05	NS
PER	7.39 ± 0.04	7.35 ± 0.05	0.003
POST	7.39 ± 0.04	7.37 ± 0.04	0.037
Cortisol (mg/dL)			
PRE	12.5 ± 5.6	14.2 ± 6.2	NS
PER	22.4 ± 5.6	22.4 ± 6.4	NS
POST	20.5 ± 8.5	27.5 ± 5.9	0.005
Insulin (μU/mL)			
PRE	4.6 ± 3.3	5.8 ± 3.7	NS
PER	3.2 ± 2.8	3.8 ± 4.5	NS
POST	7.4 ± 5.7	11.6 ± 6.3	0.02
Glucose (mg/dL)			
PRE	89 ± 9.0	78 ± 14	0.004
PER	112 ± 22	100 ± 19	NS
POST	100 ± 16.7	109 ± 20	NS
Lactate (mmol/L)			
PRE	2.2 ± 0.6	2.8 ± 0.8	NS
PER	2.8 ± 0.5	2.9 ± 0.2	NS
POST	1.8 ± 0.8	2.5 ± 0.7	NS

*PO*_*2*_ arterial oxygen pressure, *PCO*_*2*_ arterial carbon dioxide pressure, *PRE* preoperative values, *PER* peroperative (during pneumoperitonium) values, *POST* postoperative values, *NS* not significant

*p* < 0.05: statistically significant

Postoperative PaO_2_ values are higher in Group PC. Also, PaCO_2_ values during pneumoperitonium were higher and pH values during pneumoperitoneum and post-operative period were lower in Group VC. The mean post-operative cortisol and insulin levels were higher in Group VC. The mean preoperative glucose level was higher in Group VC, which was in normal limits.

## Discussion

Major finding of the present study is PCV mode was associated with lower P-peak levels before and after pneumoperitoneum and reduced systemic stress response postoperatively in patients having laparoscopic cholecystectomy. Also PCO_2_ levels during pneumoperitoneum were lower (this may be due to better gas distribution and ventilation/perfusion ratio) and postoperative PO_2_ levels were higher (this may indicate less alveolar de-recruitment) in Group PC.

In similar studies: Tyagi et al. (2011) have observed lower P-peak values and higher compliance without any changes in PaO_2_ values; Gupta et al. (2012) also reported lower P-peak levels and dead space with higher PaO_2_ in the PCV mode. In the Balick-Weber study, (2007) passage from the VCV to the PCV mode resulted in a fall in the P-peak values and an increase in the compliance without any significant differences in oxygenation, systolic and diastolic heart functions. Ogurlu et al. (2010) have reported similar findings during laparoscopic gynecological operations. In similarly designed study with the obese (Cadi et al. 2008), recorded high PaO_2_ levels with low dead space values and without significant alterations in the P-peak, P-plateau and the P-mean values in the patients ventilated with PCV mode.

The lower P-peak levels during the PCV mode have been associated most frequently with in cases of acute lung injury, acute respiratory distress syndrome, and ventilation of the single lung. Observation of higher PaO_2_ levels in the PCV mode despite the application of equal tidal volume in both the PCV and the VCV group of patients may be associated with the higher plateau and mean airway pressure recordings. However, higher oxygenation has been observed in some studies despite lack of significant differences in the airway pressure values. The improved oxygenation in the PCV mode may be attributed to ventilation with a higher flow rate and square wave pressure. The elevated flow at the beginning of inspiration causes faster rise in the pressure compared to the VCV mode and the earlier supply of a higher proportion of the tidal volume. The fast alveoli with a short time constant may be overinflated at the start of the inspiration, but the subsequent homogenization of the tidal volume spread in the alveoli prevents the incidence of the development of atalectasis. Also, despite the lowering of the inspiratory flow rate during the plateau pressure phase, the flow never reaches zero as in the VCV (Abdullah et al. 2014). The better oxygenation may be related to these properties of the PCV.

In our study, high PaCO_2_ values recorded during the pneumoperitoneum with VCV and the lower postoperative pH values were nevertheless within the physiological limits. In both group of patients, the respiratory frequency was constant. As regards the systemic stress responses, the higher postoperative glucose levels of Group VC were, again, within the physiological range. The cortisol and insulin levels were both lowered postoperatively in Group PC.

Although involving different surgery than ours, it is relevant to consider here that during the weaning from cardiopulmonary bypass in patients undergoing aorta-coronary bypass surgery, application of volume-targeted synchronized intermittent mandatory ventilation plus pressure support (SIMV + PS) or pressure-targeted bilevel positive airway pressure (BIPAP + PS) to two different group of patients did not result in significant differences in the post operative stress responses of the two groups (Calzia et al. 2001). However, it was observed that the BIPAP mode was more comfortable and the sedation requirements were reduced. Hu et al. (2014) compared the effects of VCV and pressure-controlled volume-guaranteed (PCV-VG) mode during one lung ventilation (OLV) on circulation, pulmonary function and lung injury. PCV-VG showed lower P-peak and higher compliance levels and also compared with VCV group, IL-6, and TNF-∝ of PCV-VG were significantly lower. They concluded that during OLV in thoracoscopic lobectomy, PCV-VG mode has a competitive advantage over VCV mode in terms of pulmonary function and lung protection.

The lower systemic stress response and lung injury after PCV mode usage may be due to low peak pressure, high oxygenation and compliance results lower incidence of atelectasis.

The major limitation of our study is our groups were healthy subjects. The findings of our study may not be clinically significant, but discrepancy in clinical consequences can be expected in patients with poor pulmonary function. Also, the absence of any data regarding the transpulmonary pressure may be another limitation of this study.

## Conclusion

According to our findings, when compared to VCV mode, PCV mode may improve compliance during pneumoperitoneum, improve oxygenation and reduce stress response postoperatively and may be more appropriate in patients having laparoscopic surgery.
